# Evaluation of automatic video captioning using direct assessment

**DOI:** 10.1371/journal.pone.0202789

**Published:** 2018-09-04

**Authors:** Yvette Graham, George Awad, Alan Smeaton

**Affiliations:** 1 ADAPT Centre for Digital Content Technology, Dublin City University, Glasnevin, Dublin 9, Ireland; 2 National Institute of Standards and Technology, Gaithersburg, MD, United States of America; 3 Dakota Consulting, Inc., Silver Spring, MD, United States of America; 4 Insight Centre for Data Analytics, Dublin City University, Glasnevin, Dublin 9, Ireland; UCLA, UNITED STATES

## Abstract

We present Direct Assessment, a method for manually assessing the quality of automatically-generated captions for video. Evaluating the accuracy of video captions is particularly difficult because for any given video clip there is no definitive ground truth or correct answer against which to measure. Metrics for comparing automatic video captions against a manual caption such as BLEU and METEOR, drawn from techniques used in evaluating machine translation, were used in the TRECVid video captioning task in 2016 but these are shown to have weaknesses. The work presented here brings human assessment into the evaluation by crowd sourcing how well a caption describes a video. We automatically degrade the quality of some sample captions which are assessed manually and from this we are able to rate the quality of the human assessors, a factor we take into account in the evaluation. Using data from the TRECVid video-to-text task in 2016, we show how our direct assessment method is replicable and robust and scales to where there are many caption-generation techniques to be evaluated including the TRECVid video-to-text task in 2017.

## Introduction

Describing image content precisely, either still images or video, is difficult because images are visually rich and because their content can be interpreted in so many ways. Recent years have seen huge growth in the ways in which we can access images and video online. Services like Netflix and Amazon Prime now bring moving images into our homes and onto our devices and social media services like Facebook and Twitter have created new ways in which we find this content.

Current approaches to describing images and video on the internet is by *tagging*, which involves identifying a set of objects or activities present in an image or video. Tagging is not an exhaustive process in that it does not provide complete description of content. While images and videos can be tagged manually, as a result of developments in machine learning and in particular deep learning, we can now do this automatically with good accuracy, with a wide spread of objects and activities, and with reasonable computational overhead [1].

There is a large volume of scientific activity in the multimedia community to evaluate the effectiveness of automatically generated image and video descriptions with a variety of benchmarking activities and data challenges. These include the semantic indexing task for video in TRECVid [2, 3], the detection and classification of hundreds of object categories on millions of images in ImageNet [4] and in ImageCLEF addressing the evaluation of image retrieval [5]. Benchmarking activities like these are now mainstream within the multimedia and multimedia retrieval research communities.

When it comes to describing video content, tagging becomes less attractive for video than it is for images. This is because objects in a video can appear and disappear or be occluded during the playback of a video. Thus tagging, if it was to be used, would need to be temporal and perhaps localised within video frames to show the objects that are being tagged. Tracking of tagged objects throughout a video shot might then be done based on bags of tags, one bag for each frame or object appearance. More problematic is detecting and then describing the *activities* that happen in a video, the actions and movements of objects, where simple tags are not rich or powerful enough to capture those activities or the movements. For example, temporal relationships can occur like somebody did something *and then* something else happened, or spatial relationships can be important like somebody did something while *behind them* something else happened. Such relationships are just not suited to being described by simple tags.

The alternative representation for videos is captions, descriptive narrative text which explains or describes what is happening in a video. The problem with using captions is that by its nature, videos can be so rich in terms of their content and there can be so much happening in a short video clip, that unlike image tagging where there can be a ground truth against which to measure, there is no single correct caption for a video.

In this paper we present a way to evaluate the accuracy and effectiveness of automatically-generated captions for short video clips. Our approach uses human assessments as part of the evaluation process and our work was carried out in the context of the 2016 and 2017 TRECVid Video-To-Text (VTT) pilot and full tracks respectively. The paper is organised as follows. In the next section we motivate the work by showing the need for having a human-in-the-loop for evaluations where there is no gold standard or ground truth against which to measure automatically. We then describe how in previous work we have involved human assessment in evaluations of the accuracy of machine translation. Following that, we give an overview of the TRECVid VTT task, the data used, runs submitted by participants in 2016 and metrics used while after that we describe our contribution, a method to incorporate human assessments into video captioning. Finally, in the last section we describe how we have used this human-in-the-loop for a subsequent evaluation.

## Evaluations using humans-in-the-loop

We know it is difficult to evaluate generating video captions, either manually or automatically generated, because there is no absolutely correct answer, and thus nothing to measure new systems against. This creates challenges in terms of scientific validation and replication. Scientific research demands evaluations which are reliable and reproducible and thus independently verifiable and we want to achieve this with automatic captioning so we can see which techniques and approaches work best. While this may appear to be an intractable situation, there are other areas of research where even without ground truth, evaluations can be carried out. One of those is in social media research where there is a huge surge of interest because of the importance of social media in our lives and because social media data is now easily available.

Research into social media can often have the aim of identifying some forms of behavioral patterns, such as sentiment towards issues or products, or the distribution of demographics interested in some topic, or tracking the spread, or the truthfulness, associated with news. Validating such patterns can involve tracking individual users and surveying or interacting with them. Yet social media users are scattered and generally unavailable for one-on-one interaction or for providing feedback to researchers to validate the patterns they are trying to identify or to prove.

This evaluation dilemma—how to prove research findings in a credible and reproducible way when there is no ground truth—is addressed in [6] which describes a number of techniques used in social media research. These include A/B testing such as the 2014 A/B test that Facebook carried out on almost 700,000 of its users investigating emotional contagion and the transference of emotional state [7]. It also includes spatio-temporal evaluations where predictions of future events or postings made using machine learning can be assessed by partitioning data into training and testing data. Tracking down the trigger for some event or phenomenon like a news activity or increased network traffic in some area by examining and exploring the counterfactual, namely what would have happened if the trigger event had not happened, is yet another example of a research technique that can be used when there is no ground truth.

In the case of work reported in this paper there is not a single ground truth caption for a video but instead there are many ground truths as a single video clip can have very many truthful descriptions of the video content depending on perspective, recent history, domain familiarity, even emotional state. In such an environment, asking a user to caption a video, even asking several users to caption the same video, and then measuring automatically-generated captions against these, is not robust or reliable science. What we are trying to do here is to measure the accuracy of automatically-generated video captions, some of which may be fully accurate and correct while some others will be inaccurate because the caption generation techniques are not perfect. So instead of measuring the generated captions against some pre-defined ground truth, we bring human assessment into the process and we build on parallels with human evaluations in machine translation, which we describe in the next section.

## Human evaluators in machine translation

Evaluation in many areas of natural language processing (NLP) takes inspiration from Machine Translation evaluation, including the tasks of automatic summarization [8] and grammatical error correction [9]. Evaluation in Machine Translation (MT) commonly takes the form of automatic computation of metric scores such as BLEU [10]. Here, system performance is measured as the geometric mean of matching proportions of n-gram counts between the MT output with a human produced reference translation, in addition to a penalty applied when the target translation is overly short. However, automatic MT evaluation metrics are known to provide a less than perfect substitute for human assessment, as under some circumstances, it has been shown that an improvement in BLEU is not sufficient to reflect a genuine improvement in translation quality, and in other circumstances that it is not necessary to improve BLEU in order to achieve a noticeable improvement [11]. Given the vast number of possible ways of comparing an MT output translation with a human-produced reference translation, meta-evaluation of metrics is required to determine which metrics provide the most valid substitute for human assessment. Such meta-evaluation commonly takes the form of the degree to which metrics scores correlate with human assessment. In MT, the stronger the correlation of a metric with human assessment, the better the metric is considered to be [12].

To this end, the main benchmark in MT is the Workshop on Statistical Machine Translation (WMT), which has recently changed status to a conference but retains the original acronym. At this workshop, upwards of 150 world-leading MT systems annually participate in an evaluation campaign, and where the official results comprise human evaluation of systems [13]. In WMT, a large-scale effort involving volunteer human assessors and crowd-sourced workers is carried out across several language pairs. This human evaluation not only produces official results of the translation shared task but also provides a gold standard for comparison against newly proposed automatic evaluation metrics.

Recent developments in MT have seen the development of new human assessment methodologies, one of which has been adopted as the official method of evaluation at WMT, called Direct Assessment (DA) [14]. DA involves direct estimation of the absolute quality of a given translation, in isolation from other outputs, to avoid bias introduced when translations produced by differently performing systems are compared more often to very high or to very low quality output. This would include the positive bias known to be introduced when translations belonging to a system are compared more often to low quality output [15].

In the case of MT, since genuinely bilingual human assessors are difficult and expensive to source, evaluation using DA is structured as a monolingual task, where the human evaluator is required to compare the meaning of the MT output with a human-produced reference translation, working within the same language. Assessment scores are collected on a 0–100 rating scale, which facilitates comparison of system performances based on human assessment score distributions. In addition this also allows high-quality crowd-sourcing via quality control mechanisms based on significance testing of the score distributions provided by workers. The latter is highly important for carrying out assessment via the crowd, as due to the anonymous nature of crowd-sourcing services, interference from workers attempting to game the system and to maximize their profits is unfortunately unavoidable. Even when actively rejecting assessment tasks submitted by dubious crowd-sourced workers, low quality task submission rates have been reported to be as high as between 38% and 57% [13].

The advantages of DA over previous methods of human evaluation are:

- Accurate quality control of crowd-sourcing [16];
- Absolute assessment of system performances which allows measurement of longitudinal improvements in system performance [16];
- Results for both individual translations and for systems have been shown to be almost perfectly repeatable in self-replication experiments [13, 14, 17];
- DA has been shown to be up to 10 times more effective at finding significant differences between competing systems compared to previous methodologies [18];
- DA has been shown to be efficient and cost effective for tuning automatic evaluation metrics [19].

In this paper we follow the Direct Assessment methodology used in evaluation of MT, and apply it to the monolingual task of comparing the quality of automatically generated video captions. In the next section we describe the video-to-text track in TRECVid, the data collection we used, the videos and the sets of descriptive captions generated for each, as well as the various evaluation metrics used in TRECVid.

## Video-to-text track (VTT) in TRECVid

The TREC Video Evaluation (TRECVid) benchmark has been active since 2003 in evaluating the effectiveness of content-based video retrieval systems working on problems including, but not limited to, semantic indexing, video summarization, video copy detection, multimedia event detection, and ad-hoc video search. TRECVid is organised and run each year by the National Institute of Standards and Technology (NIST) in the US. In 2016 a new showcase/pilot “Video to Text Description” (VTT) task [20] was proposed and launched within TRECVid motivated by many use case application scenarios which can greatly benefit from such technology such as video summarization in the form of natural language, facilitating the search and browsing of video archives using such descriptions, and describing videos to the blind, etc. In addition, learning video interpretation and temporal relations among events in a video will likely contribute to other computer vision tasks, such as prediction of future events from the video. The pilot VTT task from 2016 was repeated as a full task in TRECVid in 2017 [21]. In the following subsections we will review the data, task, evaluation and existing metrics used as well as the system results from participating groups.

### Data and system task

For TRECVid VTT in 2016 a dataset of more than 30 000 Twitter Vine videos had been collected automatically. Each video has a total duration of about 6s. A subset of 2 000 of these videos was randomly selected and annotated manually, twice, by two different annotators. In total, 4 non-overlapping sets of 500 videos were given to 8 annotators to generate a total of 4 000 text descriptions. Those 4 000 text descriptions were split into 2 sets corresponding to the original 2 000 videos. Annotators were asked to include and combine into 1 sentence, if appropriate and available, four facets of the video they are describing:

- Who is the video describing (e.g. concrete objects and beings, kinds of persons, animals, or things);
- What are the objects and beings doing? (generic actions, conditions/state or events);
- Where is the video taken (e.g. locale, site, place, geographic location, architectural)
- When is the video taken (e.g. time of day, season)

After annotations were completed, an automatic filtering was applied to remove very short generic descriptions which resulted in only 1 915 videos as the testing dataset.

The VTT task for participating groups was as follows: given a set of 1 915 URLs of Vine videos and two sets (A and B) of text descriptions (each composed of 1 915 descriptor sentences), participants were asked to develop systems and submit results for automatically generating for each video URL, a 1-sentence text description independently and without taking into consideration the existence of sets A and B. For distributing the video data we used the Twitter public streaming API to collect the URLs of the vine videos and we distributed the URLs. Each team downloaded their own copies of the videos and this complies with the terms and conditions for use of vine videos. The data used in the experiments in this paper is available without restriction, including a readme file to explain data format, at https://www-nlpir.nist.gov/projects/tv2016/pastdata/video.to.text/.

### Evaluation

In total, 16 individual complete “runs” were submitted to the description generation subtask. Evaluation of these was done automatically using standard metrics from machine translation (MT) including METEOR* [22] and BLEU* [10]. We add * to metric names as the way in which they were applied to captions differs in some way to how scores are produced in a standard MT evaluation. In VTT, scores were computed on the segment-level for example. BLEU (bilingual evaluation understudy) is a metric used in MT and was one of the first metrics to achieve a high correlation with human judgments of quality. It is known to perform poorly if it is used to evaluate the quality of short segments of text, like single sentences for example rather than to evaluate variants of text at a corpus level. In the VTT task in TRECVid 2016, the videos are independent thus there is no corpus to work from, so our expectations are lowered when it comes to evaluation by BLEU. METEOR (Metric for Evaluation of Translation with explicit ORdering) is based on the harmonic mean of uni-gram or n-gram precision and recall, in terms of overlap between two input sentences. It redresses some of the shortfalls of BLEU such as better matching of synonyms and stemming, though the two measures are often used together in evaluating MT.

Systems taking part in the VTT task were encouraged to take into consideration and to use the four facets that annotators used as a guideline to generate their automated descriptions.

In addition to using standard MT metrics, an experimental semantic similarity metric (STS) [23] was also applied. This automatic metric measures how semantically similar a submitted description is to the ground truth descriptions, either A or B.

### Evaluation results

**Fig 1:**
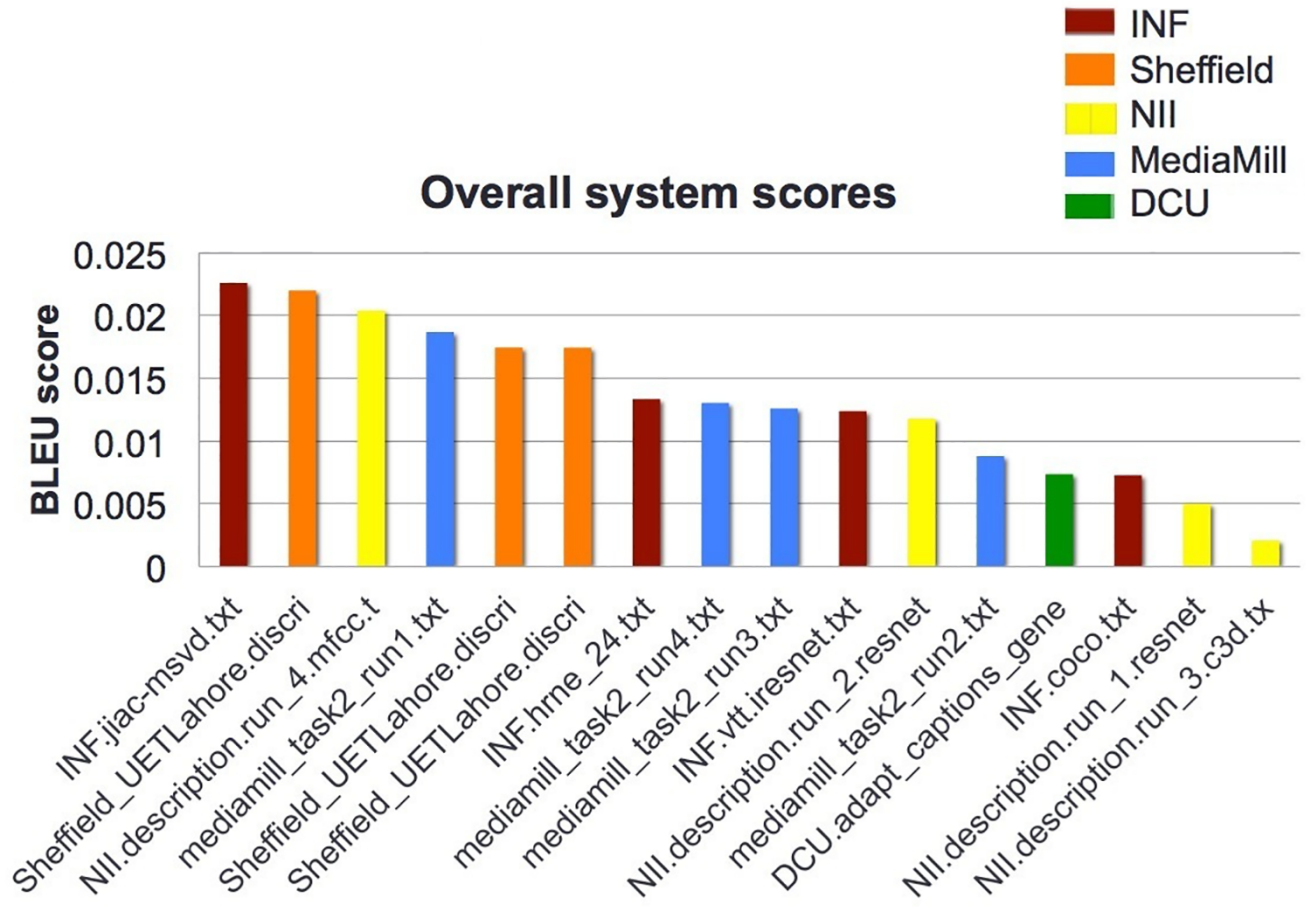
VTT: Results using the BLEU* metric (reproduced from [20], this paper is published on the TRECVID website at NIST, reviewed under the NIST Washington Editorial Review Board (WERB) internal review and is considered public domain).

**Fig 2:**
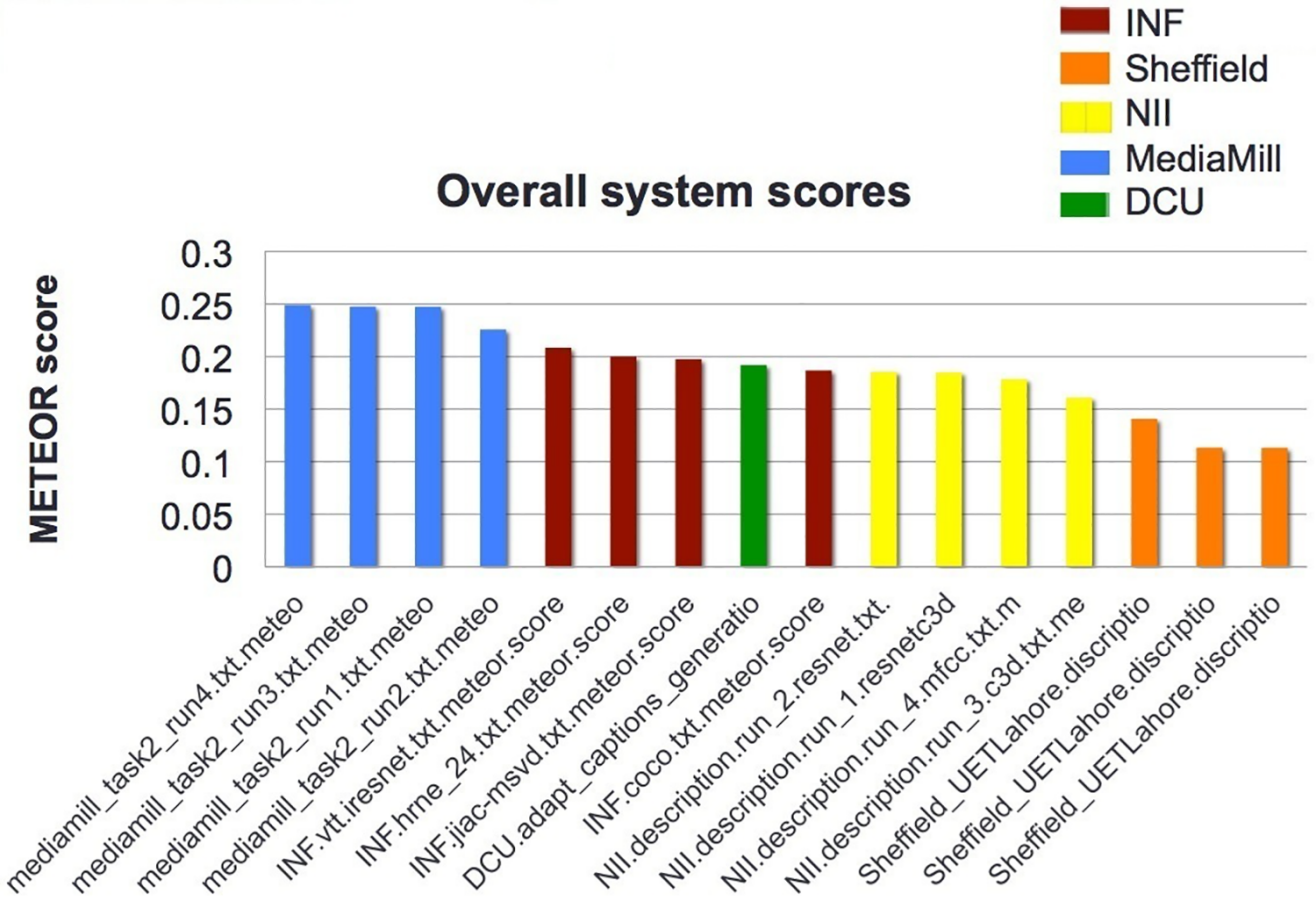
VTT: Results using the METEOR* metric (reproduced from [20]).

Figs 1 and 2 show the performance of the 16 runs submitted by the 5 participating groups using the BLEU* and METEOR* metrics respectively.

The BLEU* results in Fig 1 are difficult to interpret because, for example, multiple results from single groups are scattered throughout the results list whereas one would expect results from a single site to cluster as each group usually submits only minor variations of its own system for generating captions. This may be due to the issues associated with using BLEU* for this task, as mentioned earlier. The METEOR* results in Fig 2 show results for each group are indeed clustered by group and thus may be more reliable. However, for both BLEU* and METEOR*, trying to interpret the absolute values of the system scores is impossible so their real use is as a comparison only.

**Fig 3:**
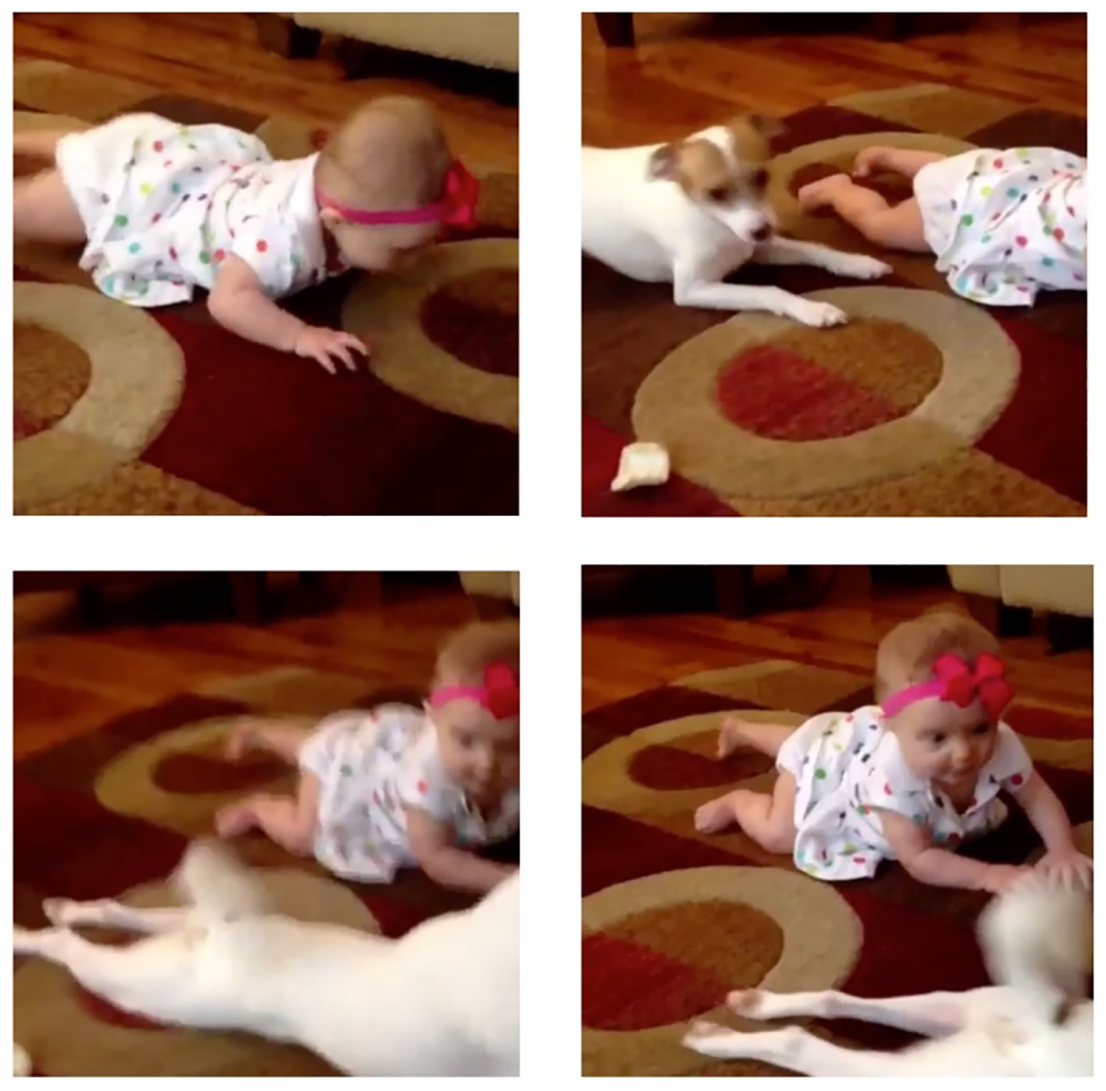
VTT: Illustrative re-enactment of sample video shown as a series of keyframes.

In order to give the reader some insight into the descriptive captions actually generated, in one of our evaluation videos a young girl on a floor, indoors, plays with a dog. For illustrative purposes we have created a video re-enactment and in Fig 3 we show some keyframes from the re-enactment. The individual in these images has given written informed consent (as outlined in PLOS consent form) to publish these images.

Below are the submitted captions for this video from across the groups (we have eliminated duplicate captions from among the 16 submissions).

- a girl is playing with a baby
- a little girl is playing with a dog
- a man is playing with a woman in a room
- a woman is playing with a baby
- a man is playing a video game and singing
- a man is talking to a car
- a toddler and a dog

What this shows is that there are good systems that do well in assigning captions, and others that do not do well in terms of the captions that they generate. Similarly there are videos which are easier to caption than others, and each approach does well on some videos and badly on others, but not consistently so. For detailed information about the approaches and results, the reader should see the various group workshop reports from the participating groups and these are MediaMill from the University of Amsterdam [24], the Informedia team from Carnegie Mellon University (INF) [25], Dublin City University (DCU) [26], the National Institute for Informatics, Japan (NII) [27], and the University of Sheffield [28].

### Semantic similarity among captions

**Fig 4:**
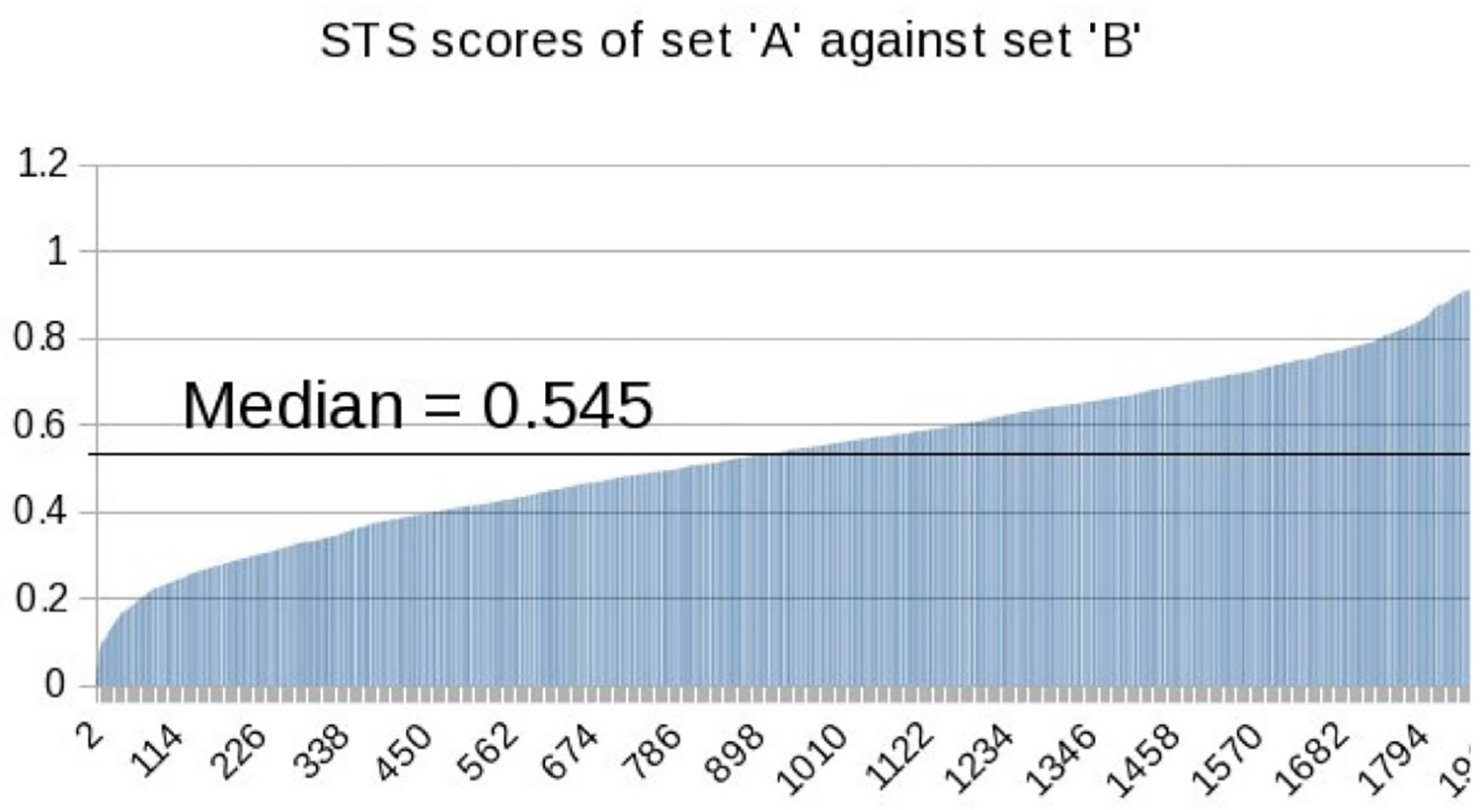
VTT: STS scores between the two reference ground truth sets (reproduced from [20]).

In addition to the BLEU* and METEOR* metrics, a semantics-based metric was also used in the VTT evaluation. This was the UMBC EBIQUITY-CORE metric [23] developed for the 2013 Semantic Textual Similarity (STS) task which uses word similarity boosted by the use of WordNet which captures word relationships. Fig 4 shows the values of the STS metric where captions A and B for each of the 1 915 videos in the evaluation are measured against each other. One would expect that the A and B captions would be semantically similar, perhaps even identical and so we would hope for a large number of the 1 915 similarity measures to be at, or close to, a value of 1.0.

Instead, as Fig 4 illustrates, the median similarity between pairs of captions (A and B) for each of the videos is only 0.545 with a disappointing tail-off of similarities close to a value of 0. That tells us that either the A and B annotators got things terribly wrong, or the STS measure has difficulty measuring similarity across just a short video caption, or the vocabulary used in the captioning creates difficulties for the STS computation. Whatever the reason, STS similarities would not add much value to interpreting the absolute performance of submitted runs though there might be some interesting insights gained from comparing relative performances.

**Fig 5:**
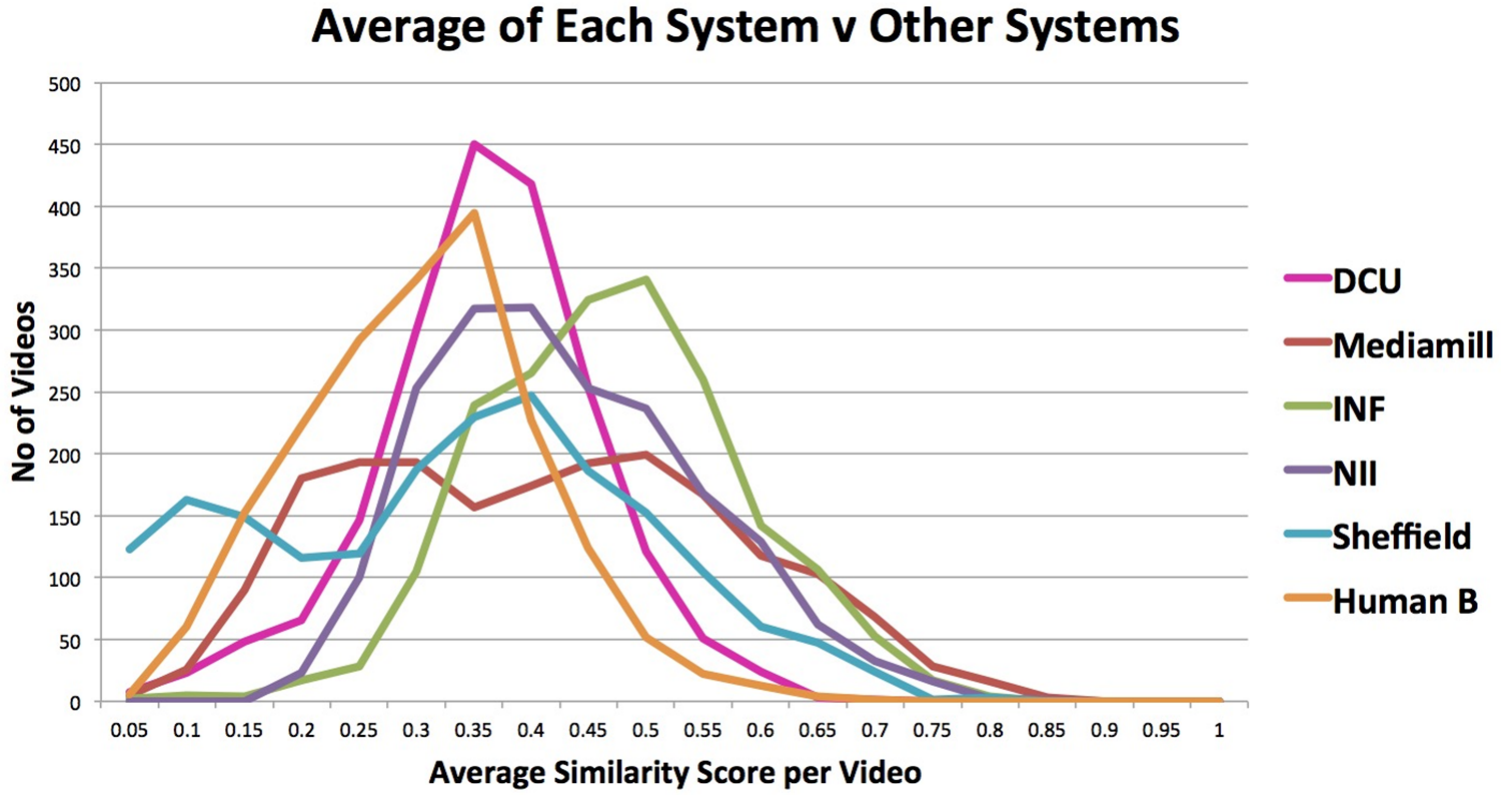
TRECVid VTT task comparing participant performance and human annotation using STS inter-system similarities.

We used an API to the UMBC STS semantic similarity score available at http://swoogle.umbc.edu/SimService/, to calculate semantic similarities among submitted captions. For the best-performing submitted run from each of the 5 groups (according to the other measures), for each video plus one of the sets of human annotations, we calculated the 6 × 6 STS similarities allowing us to see how semantically “close” or how “far” each of the submitted runs and the human annotation is to the others. Averaged over the 1 915 videos, this is shown in Fig 5.

What we observe from this analysis is that the INF group from Carnegie Mellon generates captions which are closer to the captions generated by the others including the humans than do the groups from NII, MediaMill, DCU, Sheffield, or even the human annotation, in that order. This is visible from the graph in Fig 5 where the “bulge” for each of the systems is higher and closer to a similarity score of 1.0. The line of the group from Sheffield shows an interesting kink at the very low end of the similarity scores and we interpret this as Sheffield generating some captions for some videos which are very inaccurate and thus quite dissimilar to any of the captions generated by any of the other systems or by the human annotator. From Table 1 we see that the human annotations are more dissimilar to the others than any of the automatic captions. We believe this is due to the way human captions were generated where the annotators were asked to include coverage of different facets of videos—who (is present), where (did the video take place), when (was the video taken), etc. This resulted in very structured annotations, almost formulaic in nature compared to the those generated by the automatic systems.

**Table 1:** Average STS Similarities from each system to all others.

Group	Average STS to other Groups
INF	0.446
NII	0.405
MediaMill	0.384
DCU	0.338
Sheffield	0.308
Human-b	0.279

What this analysis means that if there was a *wisdom of the crowds* type of evaluation, where the “best” performing system was the one that was close to all the others, and had fewest outliers, then the INF submission would be best. However this is evaluating caption performance from within the set of submitted runs rather than externalising the evaluation process, and this says nothing about absolute, only comparative performance.

### Summary and observations

The first observation to make about the VTT task is that there was good participation from among TRECVid groups and that there are submitted captions with impressive results. Not all generated captions are correct or impressive, but there are enough good ones to be encouraged, meaning that proper evaluation for this is really now needed.

In terms of metrics used, METEOR* scores are higher than BLEU*, and in retrospect the CIDEr metric (Consensus-based Image Description Evaluation) [29] should also have been used. As can be seen in their TRECVid workshop papers, some participating groups did include this metric in their write-ups. The STS semantic similarity metric is also useful but only insofar as it allows *comparative* performance across participating groups but such analysis does not offer any possibility for longitudinally tracking any progress over multiple iterations of the VTT task.

One aspect that became apparent as we looked at the approaches taken by different participants [26] is that there are lots of available training sets for this task, including MSR-VTT, MS-COCO, Place2, ImageNet, YouTube2Text, and MS-VD. Some of these even have manual ground truth captions generated with Mechanical Turk such as the MSR-VTT-10k dataset [30] which has 10 000 videos, is 41.2 h in duration and has 20 annotations for each video. This provides a rich landscape for those wishing to use machine learning in all its various forms within the VTT task and participants in VTT have used all of these at some point.

## Evaluating video captions

Inspired by the many advantages of Direct Assessment for evaluation of MT systems outlined earlier, we investigate the possibility of adapting DA human assessment to evaluation of video captioning. In DA, the standard set-up for MT is to present human assessors with a human-produced reference translation and an MT output in the same language and ask them to rate how well the latter expresses the meaning of the former. In DA for MT, it is necessary to create a human-produced high quality reference translation as a target of gold standard instead of the original source language input, to avoid requiring bilingual human assessors. In the case of video captioning, substitution of the video with a human-produced caption would be risky, due to the ambiguity involved in a human coming up with a caption for a video, as there are many correct but distinct captions for a single video, the meaning of which may not directly correspond to one another, unlike reference translations in MT. This risk is clearly illustrated in Fig 4 shown earlier where we see that even two manually created captions will not agree with each other when using the STS measure to assess.

Fortunately, however, substitution of the video with a human-produced caption is not only risky, it is also unnecessary, as the video can itself be included in the evaluation and viewed by the human assessors before they rate an automatically generated caption. Subsequently, we ask human assessors to firstly watch the video before reading the caption, and then assess how adequately the caption (to be evaluated) describes what took place in the video. Assessors are asked to rate the caption quality on a scale of 0 to 100.

**Fig 6:**
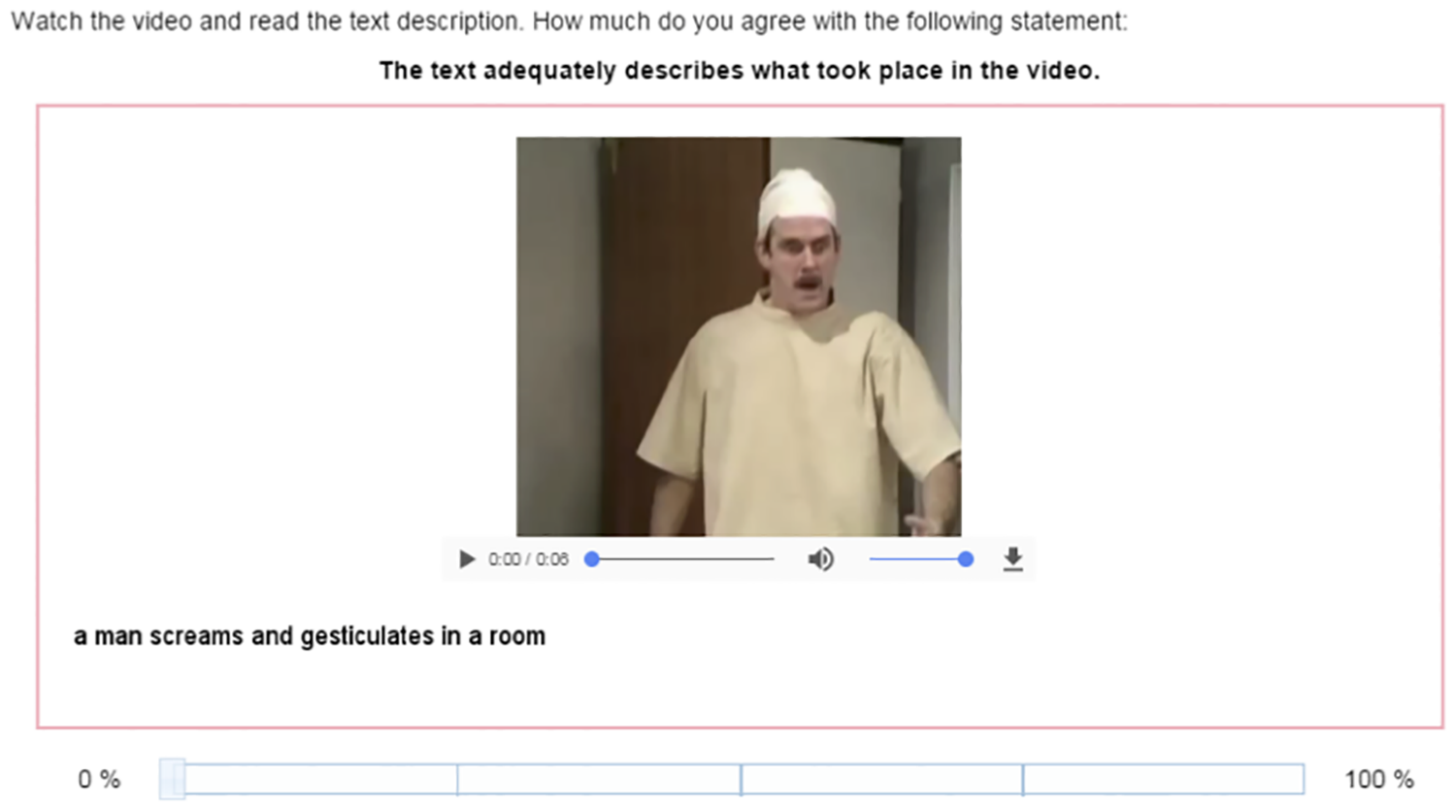
Screen shot of direct assessment of video captioning on Mechanical Turk using the same video re-enactment as earlier.

Fig 6 shows a screen shot of our DA human assessment set-up for video captioning as shown to workers on the crowd-sourcing service Amazon’s Mechanical Turk using the same video re-enactment as shown in Fig 3. Crowd sourced workers log into their Mechanical Turk account, select a work task (a HIT) to complete and begin by viewing videos and rating the caption which has been selected for them, for evaluation. Once that is completed, they then move on to the next, and the next, until they complete the task at which point they can choose a second or third or any number of assessments to complete.

### Quality controlling the crowd

As in DA for MT, we devise a mechanism for distinguishing genuine and diligent human assessors of video captions from those attempting to game the service or those who are simply not applying sufficient human intelligence to the task, by establishing the consistency of individual workers at scoring high and low quality captions for videos in the test set.

We therefore include in the evaluation of systems, an additional system comprising a set of human annotations for the video captioning test set, (A), that will act as a set of captions for which we expect to receive high scores from workers. We then create a corresponding set of low quality captions for those videos by automatically degrading each the human caption in (A). Captions are automatically degraded by randomly selecting a non-initial/non-final sequence of words within the original human caption and replacing it with a sequence of words randomly selected from the human caption of a different video. In this way, the sequence of words being substituted is a fluent sequence of words, making it difficult to spot without reading the entire caption, to help avoid further gaming approaches. The length of the phrase to be replaced is determined by the number of words in the caption to be degraded, as shown in Table 2.

**Table 2:** Substitution rules when degrading captions.

Caption Length (N)	# Words Replaced in Caption
1	1
2–5	2
6–8	3
9–15	4
16–20	5
>20	⌊ N/4 ⌋

To illustrate these substitutions, Table 3 shows some captions in their original and in their degraded forms.

**Table 3:** Sample original and degraded captions.

Original Caption	Degraded Caption
cheerleading group does throwing moves on a stage	cheerleading group moves through water in a stage
a man runs over the street and jumps into a car outdoors at daytime	a man runs over player and shoots into a car outdoors at daytime
2 men play rock paper scissors indoors at daytime	2 men wear jellabiya and dance indoors at daytime
a young man holds another one rolling on some wheels along the floor.	a young man down a small tower on some wheels along the floor.
white guy phoning someone with a syrup bottle in a room at nighttime	white guy phoning someone with a syrup dances in a living at nighttime
two white males dancing in a stadium	two white monkeys wearing in a stadium

As in MT evaluation using DA, Human Intelligence Tasks (HITs) are structured as 100-captions per HIT (or 100-translation HITs for MT) as this allows a sufficient number of pairs of high quality and low quality captions to be hidden within HITs and collected from each worker who participates. A minimum of 10 such pairs of high and low quality captions are collected from each worker and a paired significance test is then applied to the score distributions of high and low quality captions for each individual worker. The p-value produced in the test is employed as an estimate of worker reliability to score low quality captions lower than the corresponding high quality captions, with a lower p-value indicating a more consistent human assessor.

### Evaluation

In order to investigate the viability of our new video captioning human evaluation methodology, we carried out an evaluation of systems participating in TRECVid in 2016, described earlier in this paper. We include in the evaluation a single submission for each team that participated in the competition, nominated by the team from the runs they originally submitted.

All data was collected anonymously, and the study conformed to the guidelines and principles of the Declaration of Helsinki. The protocol was approved by the Dublin City University School of Computing Research Ethics Committee as it falls under the University’s category of “notification only” and was given a waiver of the requirement for signed consent because it is an anonymous online survey.

Crowd-sourced workers were paid at the same rate as in our MT experiments, at 0.99 USD per 100-caption HIT, and including the 20% Mechanical Turk fee this resulted in a total cost of 110 USD for the evaluation of five systems, or 22 USD on average per system. The cost when adding additional systems increases at a linear rate. A total of 90 workers completed at least a single 100-video caption HIT on Mechanical Turk with a relatively high proportion of those, 72 (80%), meeting our quality control requirement of a p-value less than 0.05 for significance of differences in scores they assigned to high quality and low quality captions. In addition to including high and low quality captions within HITs, we also include exact repeats of the same video and caption sampled across all systems included in the evaluation, in order to check that workers who can distinguish between the quality of high and low quality captions, also consistently score repeats of the same captions. 100% of workers who passed quality control also showed no significant difference in score distributions for repeat assessment of the same caption.

Final scores for systems are computed by firstly standardizing the raw scores provided by each worker to *z* scores which are the number of standard deviations from the mean a data point is according to that individual’s mean and standard deviation score overall. This allows any possible bias introduced by, for example, an overly harsh worker evaluating a higher number of captions from any system. Scores provided are then averaged for each caption in the test set, as some captions will have been assessed once and others multiple times, and this avoids final scores inadvertently being weighted by the number of times a given caption was assessed. The final score for a given system is then simply the average of the scores attributed to its captions.

**Fig 7:**
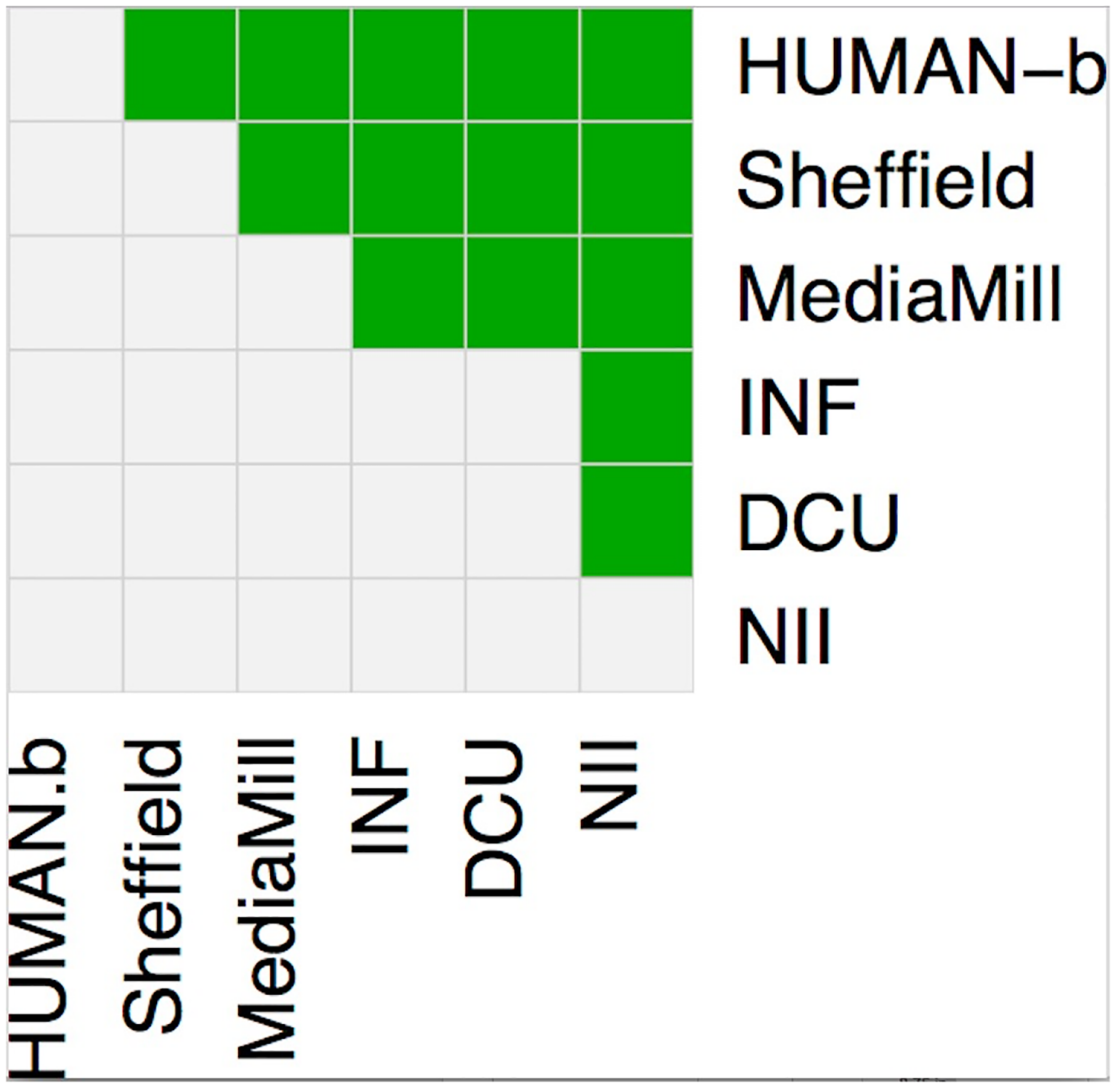
Significance test results for video captioning systems originally participating in TRECVid 2016 VTT task according to Wilcoxon rank-sum test based on *z* score distributions for systems. A green cell in a given row signifies a significant win for the system in that row over the system in that column. Human-b is a set of video captions produced by human annotators.

Table 4 shows the raw average DA scores for each system included in the evaluation, shown as a %, as well as the set of human captions, one of the sets of ground truths provided by NIST, the organisers of TRECVid, included as a hidden system, while Fig 7 shows significance test results for differences in (the more fair) *z* score distributions for systems according to Wilcoxon rank-sum test. As expected, the human captions, Human-b, achieve the highest score overall, at 88.2%, and including such a *human* system allows an estimation of the score that could be expected from a system that has effectively solved the video captioning problem. Scores for the automatic systems range from 37.7% to 66.2%, showing the best automatic system to be still some distance from human annotation quality. In terms of statistical significance, we see an almost absolute ranking of systems, with only a single pair of systems (INF & DCU) tied with no significant difference in their scores.

**Table 4:** Direct Assessment human evaluation results of nominated systems originally participating in the TRECVid 2016 VTT task; raw scores are average scores for systems; *z* scores are average standardized scores for systems; Human-b comprises one of the set of video captions produced by human annotators.

System	raw (%)	*z*	*n*
Human-b	88.2	0.895	0940
Sheffield	66.2	0.303	1,301
MediaMill	49.0	−0.129	1,292
INF	42.9	−0.278	1,338
DCU	41.3	−0.314	1,302
NII	37.7	−0.423	1,347

In order to investigate the reliability of the results produced by the new DA video captioning evaluation, we re-ran the evaluation in an entirely separate and repeated data collection/assessment on Mechanical Turk and compared results. Table 5 shows the correlation between raw average scores for systems in both the original and the repeated runs, as well as the correlation between average *z* scores for systems, the official method of system ranking we recommend. The very high correlation between scores for systems confirms that the method of evaluation is reliable, producing system rankings that are almost perfectly reproducible for evaluation of video captioning.

**Table 5:** Pearson correlation (*r*) of scores for systems produced in two separate data collection runs on Mechanical Turk.

	raw	*z*
*r*	0.995	0.997

### Metric correlation with human assessment

As mentioned previously, in MT the use of automatic metrics is validated by how well a given metric correlates with human assessment. Since we now have human assessment of captions for systems participating in TRECVid 2016 VTT, we can therefore examine how well metric scores used to produce the official results in that year’s benchmark correlate with human assessment. Table 6 shows the degree to which BLEU*, METEOR* and STS, as applied to captions in the 2016 VTT task, correlate with human assessment. To provide more detail of how metric and human scores correspond to one another, we now present respective scatter-plots of BLEU*, METEOR and STS scores and human assessment of systems.

**Table 6:** Correlation of BLEU*, METEOR* and STS scores for submissions participating in TRECVid 2016 VTT task with human assessment.

	BLEU*	METEOR*	STS	*n*
*r*	0.800	−0.602	−0.444	5

**Fig 8:**
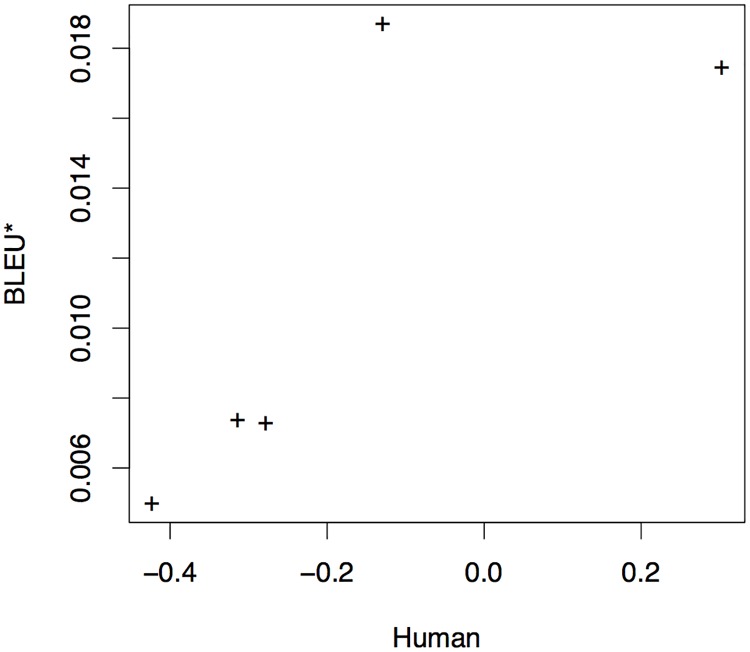
Correlation between automatic metric scores and human scoring of systems.

As can be seen in Fig 8, BLEU* scores correspond reasonably well to human evaluation, with the main disagreement taking place for the top two systems, Sheffield and MediaMill, the former outperforming the latter according to human evaluation. In contrast BLEU* scores incorrectly report a higher score for MediaMill, although only marginally so.

**Fig 9:**
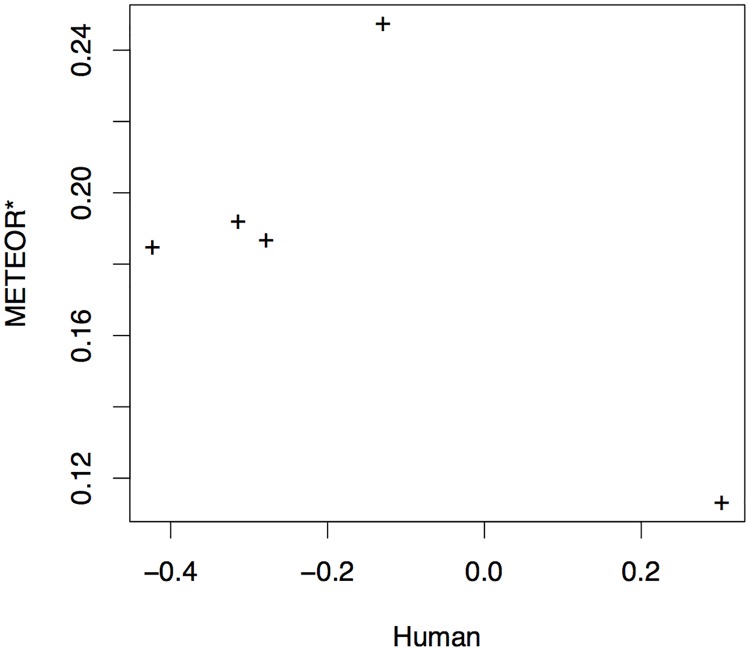
Correlation between METEOR* and human scoring of systems.

Fig 9, on the other hand, shows substantially lower agreement between METEOR* scores and human assessment compared to BLEU*, where the system scored highest according to human judges and significantly outperforming all other systems, Sheffield, is ranked worst according to this metric. This disagreement in fact results in a somewhat disconcerting *negative* correlation of METEOR* scores with human assessment of −0.602 for video captioning, as shown in Table 6, highlighting the potential danger in blindly trusting automatic metric scores and the importance of meta-evaluation by correlation with human assessment.

**Fig 10:**
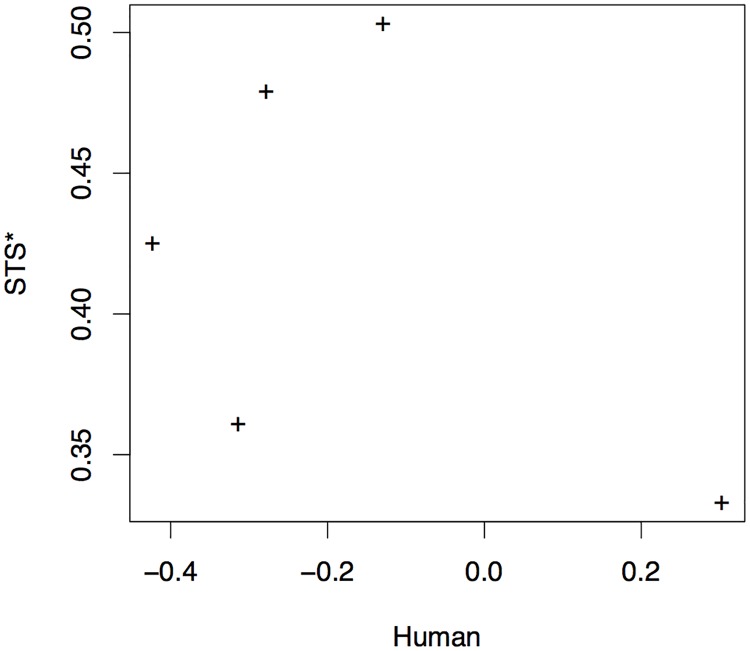
Correlation between STS and human scoring of systems.

Finally, STS scores also show low agreement with human assessment, as the scatter plot in Fig 10 shows, where, again, the best system according to human judges receives the lowest of the five STS scores.

It should be noted, however, that the correlations we report here may not provide an entirely reliable evaluation of metrics for VTT. Both the human and metric-based evaluations reported are only based on a small sample of five VTT systems, and therefore correlation point estimates are not highly reliable. Significance tests, recommended for evaluation of MT metrics and also suitable for VTT metric evaluation, such as Williams test [31, 32], do however indicate that even at this low sample size BLEU*’s correlation with human assessment is significantly higher than that of both METEOR* (at *p* < 0.01) and STS (at *p* < 0.05).

## Conclusions and future plans

In this paper we have introduced direct assessment, a method for incorporating human judgments into an evaluation of automatic video captioning. Using data from the 2016 VTT track in TRECVid we have shown how this method is robust. Even though direct assessment requires human judgments and ratings of how well a caption describes a video, this can be outsourced to a service like Mechanical Turk meaning it can be carried out in a relatively cost-effective manner.

One of the known problems with crowdsourced judgments as we use in direct assessment, is achieving consistency and quality across human judges. We address this by automatically modifying candidate captions and degrading their quality and examining how the degraded captions are rated by the human judges. This allows us to apply quality control to the assessors meaning that direct assessment can be replicated consistently, something we demonstrated in the paper.

As part of the evaluation of performance in the VTT track in TRECVid 2017, we used the DA method in addition to BLEU, METEOR and the other standard metrics with an increased number of groups participating as described in [21]. We used the nominated runs submitted by each team in the DA assessment and the turnaround time between participants submitting runs and the DA-based assessment results being available, was about 8 days in total, which include data preparation and cleaning, submission to Mechanical Turk, crowd sourced assessment, results gathering and results computation.
